# Comparison of sliding window and field-in-field techniques for tangential whole breast irradiation using the Halcyon and Synergy Agility systems

**DOI:** 10.1186/s13014-021-01942-y

**Published:** 2021-11-06

**Authors:** Anne Richter, Sonja Wegener, Kathrin Breuer, Gary Razinskas, Stefan Weick, Florian Exner, Klaus Bratengeier, Michael Flentje, Otto Sauer, Bülent Polat

**Affiliations:** Department of Radiation Oncology, University of Wuerzburg, Wuerzburg, Germany

**Keywords:** Whole breast irradiation, Halcyon, IGRT, Dose to OARs

## Abstract

**Background:**

To implement a tangential treatment technique for whole breast irradiation using the Varian Halcyon and to compare it with Elekta Synergy Agility plans.

**Methods:**

For 20 patients two comparable treatment plans with respect to dose coverage and normal tissue sparing were generated. Tangential field-in-field treatment plans (Pinnacle/Synergy) were replanned using the sliding window technique (Eclipse/Halcyon). Plan specific QA was performed using the portal Dosimetry and the ArcCHECK phantom. Imaging and treatment dose were evaluated for treatment delivery on both systems using a modified CIRS Phantom.

**Results:**

The mean number of monitor units for a fraction dose of 2.67 Gy was 515 MUs and 260 MUs for Halcyon and Synergy Agility plans, respectively. The homogeneity index and dose coverage were similar for both treatment units. The plan specific QA showed good agreement between measured and calculated plans. All Halcyon plans passed portal dosimetry QA (3%/2 mm) with 100% points passing and ArcCheck QA (3%/2 mm) with 99.5%. Measurement of the cumulated treatment and imaging dose with the CIRS phantom resulted in lower dose to the contralateral breast for the Halcyon plans.

**Conclusions:**

For the Varian Halcyon a plan quality similar to the Elekta Synergy device was achieved. For the Halcyon plans the dose contribution from the treatment fields to the contralateral breast was even lower due to less interleaf transmission of the Halcyon MLC and a lower contribution of scattered dose from the collimator system.

## Background

Breast cancer is one of the most common malignant tumors in women. Standard treatment for early-stage breast cancer is breast-conserving surgery followed by adjuvant whole breast irradiation [1].

The sparing of the organs at risk is important as the long term survival probability for diagnosed stage I and II patients treated with radiation after breast‐conserving therapy is high [2, 3]. Different treatment modalities are available for radiation delivery like three dimensional conformal therapy (3D‐CRT) [4, 5], tomotherapy [6–9], step-and-shoot or dynamic sliding window intensity‐modulated radiation therapy (IMRT) and volumetric modulated arc therapy (VMAT) [9–11].

The Varian Halcyon linac offers 6 MV flattening-filter-free (FFF) photon beam energy with a dual-layer multi-leaf collimator (MLC). Treatment on the Halcyon requires daily image-guidance because there are no light field, optical distance indicator or lasers at the treatment isocenter available.

The first aim of this work was to implement a treatment technique suitable for tangential whole breast irradiation using the Halcyon – with similar plan quality compared to Synergy Agility. Second aim was to investigate the dosimetric effect of daily imaging on the Halcyon unit for the contralateral breast. A comparison of the Halcyon and Synergy Agility plans in terms of imaging dose and deliverability was performed by QA measurements.

## Methods

### Patient characteristics

All patient characteristics are listed in Table 1. The patients were treated via tangential whole breast irradiation without supraclavicular or internal mammary lymph nodes. The size of the planning target volumes ranged from 351 cm^3^ to 1439 cm^3^ with a mean target volume of 814 cm^3^. The cohort consisted of two subgroups. The first ten patients were treated with Elekta Synergy Agility (Elekta Medical Systems, Crawley, UK). Treatment plans with tangential beams were re-planned for Halcyon to compare the plan quality and dose to OARs retrospectively. The second subgroup of 10 patients was scheduled for whole breast irradiation on the Varian Halcyon system (Varian Medical Systems, Palo Alto, CA, USA, version 2.0) while treatment plans for Halcyon and Synergy Agility were created. Finally, two comparable plans were available for all patients.

**Table 1:** Patient and treatment characteristics

Treatment machine	Patient	Tumor site	PTV in cm^3^	Energy		Fields		Monitor Units	
				Synergy	Halcyon	Synergy	Halcyon	Synergy	Halcyon
Synergy Agility	1	Left	690	6 & 10MV	6MV FFF	4	2	245	446
	2	Left	1163	6MV	6MV FFF	4	2	268	499
	3	Left	832	6MV	6MV FFF	4	2	261	502
	4	Left	946	6MV	6MV FFF	4	2	263	440
	5	Left	435	6MV	6MV FFF	4	2	253	436
	6	Right	790	6MV	6MV FFF	4	2	256	470
	7	Right	509	6 & 10MV	6MV FFF	5	2	254	470
	8	Right	934	6MV	6MV FFF	4	2	269	494
	9	Right	351	6 & 10MV	6MV FFF	5	2	263	492
	10	Right	1220	6MV	6MV FFF	4	3	274	700
Halcyon	11	Left	1016	6MV	6MV FFF	4	2	277	536
	12	Left	798	6MV	6MV FFF	4	2	259	415
	13	Left	589	6MV	6MV FFF	4	2	266	575
	14	Left	861	6MV	6MV FFF	4	2	254	484
	15	Left	1035	6MV	6MV FFF	4	2	254	641
	16	Right	1439	6MV	6MV FFF	4	2	266	536
	17	Right	494	6MV	6MV FFF	4	2	243	459
	18	Right	496	6MV	6MV FFF	4	2	246	596
	19	Right	879	6MV	6MV FFF	5	2	274	495
	20	Right	792	6MV	6MV FFF	4	2	251	618
	Mean		813.5			4.2	2.1	259.8	515.2
	SD		283.5			0.4	0.2	10.1	75.6

Patient characteristics and details of the beam configuration, energy and segments used. Stated values indicate mean ± SD. Patient #1–10 were treated on the Synergy Agility machine and patients #11–20 were treated on the Halcyon system

### Treatment planning

Patients were positioned in supine position with both arms raised. CT imaging and treatment were performed in free breathing. The dose prescription was 40 Gy in 15 fractions followed by a boost of 10 Gy in 5 fractions (mean dose). PTV was delineated closed to the surface. Dose homogeneity was maintained by the standard deviation (SD) in PTV-5 mm of less than 3%. PTV-0.5 mm corresponds to the CTV as defined in the ESTRO Guideline [12, 13].

Pinnacle (Philips Radiation Oncology Systems, Fitchburg, WI, version 16.2) was used for treatment planning of the Elekta Synergy Agility plans. A 3D-CRT field in field technique was used with flattened 6 MV and 10 MV beams in forward planning mode [4]. The gantry angle was optimized in the beam’s eye view (BEV) for a minimum lung area and beam divergence toward the lung was compensated by adjusting the gantry angles of the beams accordingly. The beams exceeded the patient surface by minimum of 2 cm. One to two segments per field were added to improve target coverage and dose homogeneity and to omit wedges. This procedure of treatment planning for the Synergy platform resulted in two tangential beams and one to three additional segments with the same tangential gantry angles (4 – 5 beams in total, see Table 1). Final dose was calculated using the collapsed cone algorithm and a grid size of 0.3 cm.

Treatment planning for the Halcyon system was performed in Eclipse (Varian Medical Systems, Palo Alto, CA, USA, version 15.6). Tangential fields were defined using 6 MV FFF beams. The dose distribution was optimized with a sliding window technique (IMRT). A virtual bolus was added on top of the PTV during the optimization process with a density of 0.26 g/cm^3^ and a thickness of 2 cm. With a minimum dose objective for the virtual bolus the optimizer was forced to enlarge the field size into the air. An uncertainty analysis was carried out by shifting the isocenter (1 cm) in dorsal and contralateral direction which should result in comparable target coverage for PTV-5 mm. For final dose calculation, the bolus was removed. No bolus was used during treatment. The same tangential beam arrangement was used for the Halcyon and Synergy Agility plans. For Halcyon, no additional segment fields were needed due to the sliding window technique which resulted in 2–3 beams (see Table 1). No couch rotation was utilized in the clinical plans for any of the patients selected. In Eclipse, dose was calculated with a grid size of 0.25 cm using the Acuros External Beam Algorithm, version 15.6.06.

### Treatment plan comparison

We compared the dose-volume histograms (DVHs) of the Halcyon with the Synergy Agility plans. DVH parameters are listed in Table 2. For PTV, the dose received by 2%, 50%, 80%, 95% and 98% volume (D02, D50, D80, D95, D98) were evaluated. The homogeneity index (HI) was defined as HI = (D02—D98) / D50. For OARs, the maximum and mean doses were assessed. The number of monitor units (MUs) and the correlation between PTV size and number of MU were analysed for all plans. Both machines (Synergy Agility and Varian Halcyon) were calibrated equally: a dose of 1 Gy in 10 cm depth corresponds to 100 MU.

**Table 2:** DVH comparison between Synergy and Halcyon plans

	Synergy Agility	Halcyon	*p* value
**PTV**			
D95 in Gy	33.9 ± 2.3	33.5 ± 2.5	NS*
D80 in Gy	38.0 ± 0.5	38.6 ± 0.4	*p* < 0.01
D_Max_ in Gy	43.0 ± 0.6	43.3 ± 0.8	NS
HI	0.13 ± 0.01	0.12 ± 0.02	NS
**PTV-0.5**			
D_Min_ in Gy	35.3 ± 1.0	35.4 ± 0.2	NS
D95 in Gy	38.5 ± 0.2	38.8 ± 0.3	0.03
D_Mean_ in Gy	40.1 ± 0.1	40.1 ± 0.1	NS
SD in Gy	1.0 ± 0.1	0.8 ± 0.1	0.01
**Lung ipsilateral**			
D_Mean_ in Gy	4.7 ± 0.9	4.4 ± 1.1	NS
**Lung contralateral**			
D_Mean_ in Gy	0.3 ± 0.1	0.2 ± 0.1	*p* < 0.01
**Breast contralateral**			
D_Mean_ in Gy	0.5 ± 0.2	0.3 ± 0.1	*p* < 0.01
D01 in Gy	1.8 ± 0.7	1.0 ± 0.5	*p* < 0.01
Heart**			
D_Max_ in Gy	31.7 ± 10.5	33.0 ± 8.4	NS
D_Mean_ in Gy	1.7 ± 0.4	1.5 ± 0.5	*p* < 0.01

Dose to target volumes and organs at risk in Synergy Agility plans and Halcyon plans. Stated values indicate mean ± SD and statistical analysis was performed using the Wilcoxon test. The differences were considered statistically significant when *p* < 0.05

^*^Not significant (NS)

^**^Only left-sided plans were considered for the evaluation of the heart dose

### Dosimetric evaluation

#### Plan specific QA

In order to test the deliverability of the Halcyon treatment plans, plan specific QA was performed. Plans were delivered to an ArcCHECK Phantom (Sun Nuclear Corporation, Melbourne, FL, USA) with the cavity plug present equipped with a Semiflex 31,010 ionization chamber (PTW-Freiburg, Freiburg, Germany) in the center. For point dose measurement, passing criteria were percent dose deviation of less than 3% from the expected dose. The ArcCHECK measurements were evaluated with 3%/2 mm and 2%/2 mm gamma criteria with tolerance level of 96.5% and 95% of pixel passing, global normalization and 10% low dose threshold. Portal dosimetry was evaluated for each field with a gammy criterion of 2%/1 mm and a tolerable passing rate of 94%.

### Imaging and treatment dose

**Fig. 1:**
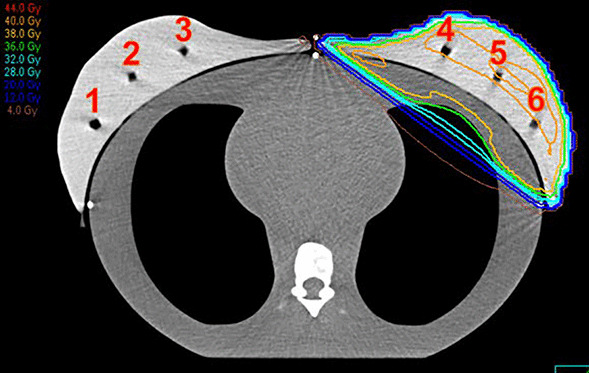
CIRS phantom with breast surrogates. Measurement of imaging and treatment dose was performed using a CIRS phantom with breast surrogates. Point dose was measured for the contralateral (1–3) and ipsilateral side (4–6). The treatment plan for the Synergy Agility is illustrated

To evaluate the imaging and treatment dose a left-sided treatment plan was generated for a dynamic thorax phantom (CIRS Incorporated, Norfolk, Virginia, USA). The phantom was used in static mode. Breast surrogates were attached to simulate a breast treatment. Breast surrogates were created from silicon material with 3 inserts for an ionization chamber for each side (see Fig. 1). A Unidos dosimeter (PTW-Freiburg, Freiburg, Germany) with 0.3 cm^3^ thimble chamber (type TM31013) was used for dose measurements. The dosimetric influence of different imaging modes (MV planar imaging, volumetric kV CBCT) was compared by measuring the dose in the ipsilateral and contralateral breast. In total, six point dose values were acquired for each imaging setup. For the Halcyon CBCT system, the breast mode with 125 kV and 45 mAs was used followed by iterative image reconstruction. This reconstruction algorithm is designed to reduce noise and to enhance image quality with high resolution. MV portal imaging is limited to 0° and 90° gantry position for the Halcyon system. For the Synergy Agility treatment setup, imaging dose of kV CBCT (120 kV, 70.4 mAs, S20) was compared with MV images at 0° gantry position followed by the tangential treatment angles.

For this anthropomorphic breast phantom, treatment plans were optimized for both the Synergy Agility and Halcyon machines. For our standard protocol, imaging and treatment doses were measured and the accumulated dose in the contralateral breast was determined. A typical imaging procedure on the Synergy Agility system would be to perform imaging with anterior–posterior and tangential MV portals before the first treatment followed by imaging once a week (3 fractions of MV imaging and 15 treatment fractions). In contrast, the treatment on the Halcyon system required imaging before each treatment fraction (15 CBCT imaging fractions and 15 treatment fractions).

### Statistical analysis

A statistical comparison of the Halcyon and the Synergy Agility plans was implemented to analyze dosimetric differences between the two machines using the Wilcoxon test for bound comparisons. The differences were considered statistically significant when *p* < 0.05. IBM SPSS Statistics for Windows, version 26 (IBM Corp., Armonk, N.Y., USA) was used for statistical evaluation.

## Results

### Treatment plan comparison

The beam configuration for Halcyon and Synergy Agility plans are listed in Table 1. For most cases, 6 MV and 4 fields were sufficient for the field-in-field technique with the Synergy Agility. In 3 out of 20 cases, a field arrangement of 5 beams was needed. A mixture of 6MV and 10MV was used in 3 cases. For treatment planning on the Halcyon unit, two treatment fields were used for application of a sliding window technique. Just for one case 3 fields were applied.

Table 1 outlines the difference in MUs for the Halcyon vs. Synergy Agility plans. The mean number of MUs used for the Halcyon delivered plans was 515 MUs, whereas the Synergy Agility delivered plans used 260 MUs (*p* < 0.001). No significant correlation was found between PTV size and number of MUs (R^2^ = 0.5 and R^2^ = 0.1 for Halcyon and Synergy Agility plans, respectively).

Overall, the plan quality of Halcyon plans was comparable to plans delivered on Elekta Synergy linac. Mean dose values in the PTV and OARs are listed in Table 2. For both treatment units the homogeneity index (HI) was similar: Synergy Agility plans achieved HI of 0.13 while the Halcyon plans achieved 0.12. Slightly improved dose homogeneity was observed for the Halcyon plans. Only the D80 in PTV differed significantly by 0.6 Gy on average (*p* < 0.01). The SD in PTV-5 mm was 1.0 Gy and 0.8 Gy on average for Synergy Agility and Halcyon plans, respectively. Significant differences were observed for D95 and SD in PTV-5 mm. However, given that dose differences are very low, we do not consider them of clinical relevance. Nominal target coverage (D95 and D80) was comparable for Halcyon and Synergy Agility plans.

Mean dose in the lungs and heart was slightly improved for Halcyon plans. Significant differences were found for the mean dose in the contralateral lung (0.1 Gy), the heart (0.2 Gy) and the contralateral breast (0.2). Maximum dose in the heart was 1.3 Gy lower on average for Synergy Agility plans but not statistically significant.

### Plan specific QA

Phantom measurements with the ArcCHECK and portal dosimetry were performed for the Halcyon plans. All plans were successfully delivered on the Halcyon unit. Table 3 shows the ion chamber measurements and gamma passing rates for all patient plans. For the ArcCHECK, the mean deviation of the ion chamber dose was − 1% with a maximum deviation of − 2.7%. The pass rate of the diode measurements was 99.5% for the 3%/2 mm gamma criterion.

**Table 3:** Plan specific QA results

Patient	Ion chamber dose deviation in %	Portal dosimetry		ArcCHECK	
		Field #1 2%/1 mm > 94%	Field #2 2%/1 mm > 94%	3%/2 mm > 96,5%	2%/2 mm > 95%
1	− 2.7	99.3	96.6	100	100
2	− 1.6	99.5	96.2	100	98.3
3	− 1.6	99.3	96	100	98.6
4	− 1.5	99.5	97.1	99.8	97.2
5	− 1.9	99.3	96.5	99.7	96.9
6	− 1.3	96.8	98	100	98.8
7	− 1.2	95.3	97.6	99.7	96.8
8	− 1.0	97.7	98.1	100	99.1
9	− 1.3	96.0	97.5	100	99.6
10	− 1.3	96.7	97.2	98.4	92.2
11	− 1.2	98.4	97.5	97.6	95.5
12	− 1.2	99.5	97.3	99.5	95.1
13	− 1.0	98.9	97.4	100	98.9
14	− 0.8	98.9	96.6	99.8	96.5
15	− 1.1	99.2	94.6	98.9	92
16	− 1.6	97.8	96	97.6	88.3
17	− 1.6	94.8	97.2	99.7	97.8
18	2.5	94.6	95.8	98.5	95.8
19	2.4	97.1	97.5	99.8	99
20	− 0.5	97.2	95.8	100	99.2
Mean	− 1.0	97.8	96.7	99.5	96.8
SD	1.3	1.7	0.9	0.8	3.0

Results of the plan specific QA for ion chamber measurement, portal dosimetry and the ArcCHECK phantom

All portal dosimetry measurements passed the 3%/2 mm gamma criterion with 100% of the pixels. The evaluation of the 2%/1 mm gamma criterion resulted in a mean pass rate of 97.8% and 96.7% for field #1 and field #2, respectively.

### Imaging and treatment dose

**Fig. 2:**
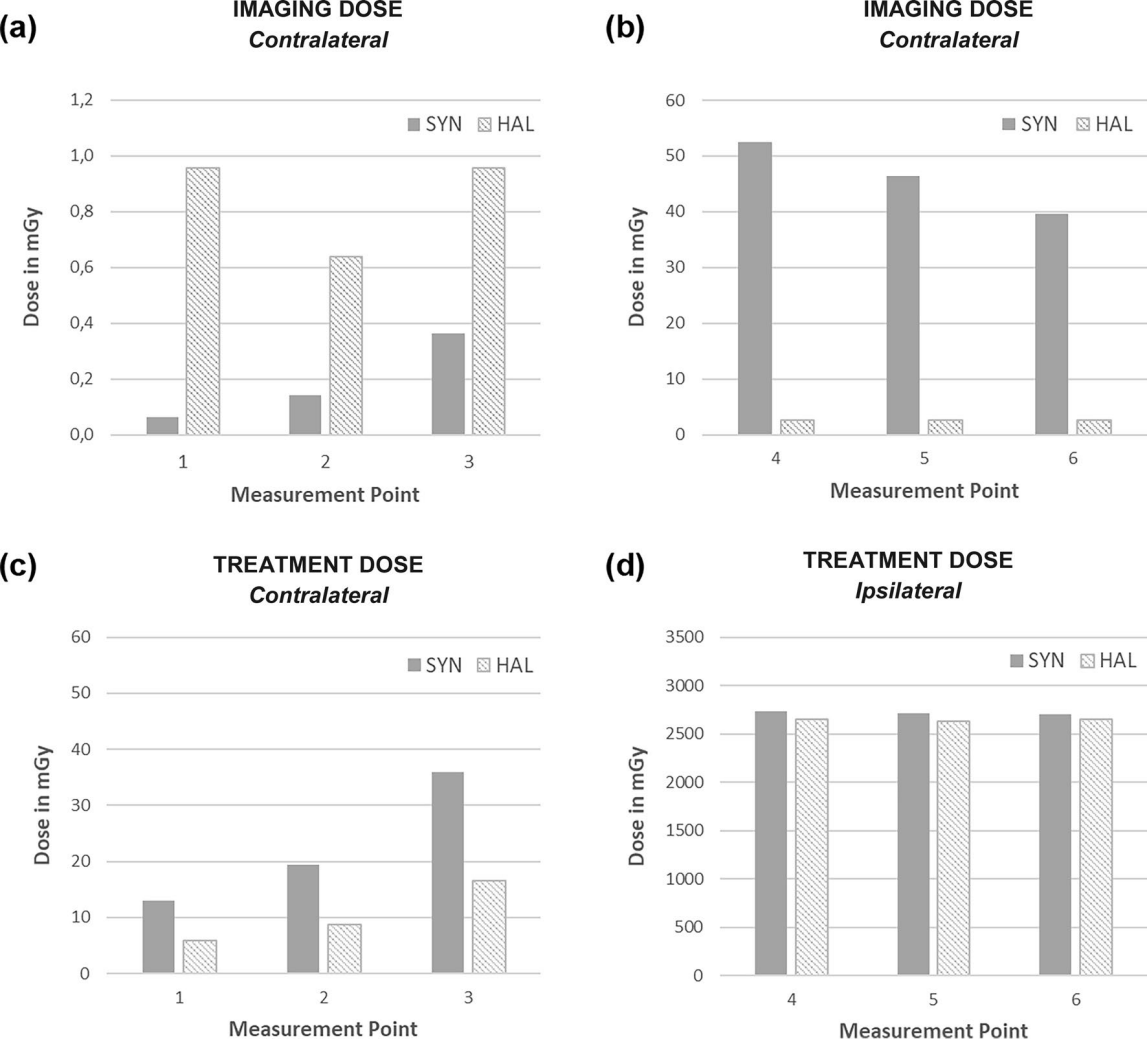
Imaging and treatment dose. Dose contribution by imaging with the standard protocol (**a**, **b**) and by one treatment fraction (**c**, **d**) is displayed for the Synergy Agility (grey) and Halcyon unit (striped area). Results for the contralateral breast (points 1–3) are plotted on the left (**a**, **c**). The results for the ipsilateral breast (points 4–6) are shown at the right (**b**, **d**)

For treatment setup on a Synergy Agility unit, the imaging dose of MV portals was compared with kV CBCT. A summary of all measurements is given in Table 4 and Fig. 2. The lowest imaging dose in the contralateral breast (points 1–3) was measured for the anterior–posterior and tangential MV portals on the Synergy Agility (in total 6 MU). Dose in points 1, 2 and 3 was 0.1 mGy, 0.1 mGy, and 0.4 mGy, respectively. Slightly larger doses were measured for the kV CBCT setup with 0.1 mGy, 0.2 mGy and 0.8 mGy in points 1–3. The cumulated dose of all treatment and imaging fractions for the Synergy Agility plan would result in 195.2 mGy, 291.8 mGy and 541.1 mGy in points 1, 2 and 3 of the contralateral breast, respectively (Table 4). Dose was accumulated for 3 fractions of MV imaging and 15 treatment fractions.

**Table 4:** Imaging and treatment dose

		Dose in mGy					
		Contralateral side			Ipsilateral side		
		1	2	3	4	5	6
Synergy Agility	kV CBCT	0.1	0.2	0.8	1.9	2.0	2.9
	MV portals*	0.1	0.1	0.4	52.5	46.5	39.7
	Treatment	13.0	19.4	36.0	2737.1	2711.8	2704.2
	15 fx treatment	195.0	291.3	540.0	41,056.5	40,677.2	40,563.4
	3 fx imaging	0.2	0.4	1.1	157.5	139.4	119.1
	Total	195.2	291.8	541.1	41,213.9	40,816.6	40,682.5
Halcyon	kV CBCT*	1.0	0.6	1.0	2.6	2.6	2.6
	MV portals	3.5	3.2	3.8	23.9	25.8	24.5
	Treatment	5.9	8.8	16.5	2649.2	2634.7	2645.0
	15 fx treatment	88.0	131.6	247.5	39,737.7	39,519.8	39,675.4
	15 fx imaging	14.3	9.6	14.3	38.3	38.3	38.3
	Total	102.3	141.1	261.9	39,776.0	39,558.0	39,713.7

Dose was measured for the ipsilateral and contralateral side in the CIRS phantom for kV and MV imaging procedures and one treatment fraction. Total dose for our standard imaging protocols (marked with *) and all treatment fractions is listed

For treatment setup on the Halcyon unit, the imaging dose of MV portals was compared with the kV CBCT. The MV portals deliver more dose to the contralateral breast than the kV CBCT. For MV portal imaging from 0° and 90° gantry angle, dose measurement in points 1–3 resulted in 3.5 mGy, 3.2 mGy and 3.8 mGy respectively. The imaging dose of the Halcyon kV CBCT is comparable with the Synergy Agility CBCT. Dose values in points 1–3 was 1.0 mGy, 0.6 mGy and 1.0 mGy respectively. For treatment on the Halcyon unit setup imaging must be performed daily. The cumulated dose of all treatment and imaging fractions for the Halcyon plan would result in lower doses with 102.3 mGy, 141.1 mGy and 261.9 mGy in points 1, 2 and 3 of the contralateral breast, respectively (Table 4). In this case, dose was accumulated for 15 fractions of CBCT imaging and 15 treatment fractions. Thus, our standard approach with weekly MV imaging at the Synergy linac resulted in a higher dose for the contralateral breast than the daily kV imaging at the Halcyon linac.

## Discussion

The presented work shows that the Halcyon linac is suitable for whole‐breast irradiation using a tangential beam technique. A practical and efficient planning method for delivering 3D conformal breast radiotherapy using the Halcyon was implemented in our department. The simplified workflow on the Halcyon system enables a fast delivery with a reduced probability of patient collisions due to the closed ring gantry design and collision warning during treatment planning.

The overall plan quality regarding target volumes and OARs was comparable for plans delivered on Halcyon and Synergy Agility machines. Homogeneity index was 0.13 and 0.12 for Synergy and Halcyon plans, respectively. Similar results for the comparison of plan quality of C‐arm and Halcyon plans were reported by other groups [14, 15]. Flores et al. used the irregular surface compensator technique and compared to the results of the current study reported lower homogeneity indices of 0.09 and 0.08 for C-arm and Halcyon linacs, respectively [14]. The increased number of MUs for delivering the Halcyon plans was also observed by other researchers [14–16]. For the current investigation, the MUs were 50% higher than the field-in-field breast treatment plans using tangential beams with Synergy Agility. The reason is the sliding window technique in combination with the un-flattened beam for the Halcyon plans.

In the current work, lower mean doses in the OARs were observed for the Halcyon plans, while the maximum heart dose was on average 1.3 Gy higher for the Halcyon plans. In contrast, Flores et al. and Morris et al. observed an increase in the mean dose in the contralateral lung and the heart for the Halcyon plans compared to C-arm linacs [14, 15].

Dose to OARs needs to be considered when choosing the setup imaging technique (MV portals or CBCT). Comparable imaging dose for kV CBCT was observed for both systems in the current work. However, the Halcyon requires CBCT imaging to be performed at every fraction. Regarding the sum of imaging and treatment dose for our standard protocol, much lower doses to the contralateral breast were determined with the Halcyon unit. This is mainly due to the lower interleaf transmission for the Halcyon MLC and a contribution of scattered dose from the collimator system. Additional low dose is caused by the larger number of beams for the Synergy plans (4–5 beams) compared to Halcyon plans (2–3 beams). The Halcyon MLC has 77 mm thick leaves in each bank, creating a combined thickness of 154 mm. The combined transmission through the dual layer MLC is less than 0.01% at 6 MV FFF [15, 17]. Whereas for the Agility MLC the transmission through the MLC is 0.44% at 6 MV and 0.52% at 10 MV [18]. Flores et al. suggested a dose reduction for the OARs by using angled MV portals or non-ionizing techniques like surface guided RT [14].

The plan specific QA indicate a good agreement between the measured and calculated dose. The point dose measurements in the ArcCHECK phantom showed an average and maximum deviation of − 1% and − 2.7% respectively. Similar findings were reported by Morris et al. with an average and maximum point dose deviation of 1.5% and 2.23% in a solid water phantom [15]. In the current work, the average ArcCHECK gamma passing rate was 99.5% for 3%/2 mm criterion. Morris et al. published high pass rates of 99.4% for plan specific QA with Delta4 [15]. Portal dosimetry yielded pass rates of 100% for the 3%/2 mm criterion which is consistent with the results published by Morris et al. [15]. In order to get some variation the 2%/1 mm gamma criterion was used, still resulting in mean gamma pass rates above 96%.

A common technique to spare the heart for left‐sided treatments is deep inspiration breath‐hold (DIBH). Barsky et al. reported about initial experience in treating patients with breast cancer on a Halcyon system in either supine position, with or without DIBH, or prone position. DIBH was used in 29% of the patients with average treatment times of 5.8 min [19]. Kennedy stated the total treatment time can be drastically reduced using irregular surface compensator technique as opposed to the Dynamic Flattening Beam-enabled field-in-field technique [16].

A bolus is commonly applied to improve target coverage near the surface while also enhancing the risk of severe skin reactions and a possible negative impact on cosmesis. The effect on superficial dose for 6 MV FFF beams and the need for bolus was evaluated by O’Grady et al. [20]. An increased superficial dose was observed for the use of a Halcyon 6MV FFF beam compared to 6 MV beams on C-Arm linacs for whole breast irradiation. It was assumed by O’ Grady et al. that the increased dose is primarily caused by a higher proportion of low energy photons. The traditional application of a bolus should be carefully re-evaluated for 6 MV beams [20].

In the current work, we only investigated tangential beam arrangements. Several modalities are available for whole breast irradiation, e.g., the Irregular Surface Compensation technique and dynamic flatting beam sequences. For target volumes including the mammaria interna lymph nodes, we currently use VMAT and multi field IMRTs (consisting of 13 to 19 beams) on Synergy Platforms. The possibilities of treating such cases with the Halcyon will be investigated in a subsequent examination.

The field size of the Halcyon system is limited to 28 × 28 cm^2^. A multi isocenter technique allows an extension up to a field size of 36 cm in longitudinal direction with a maximum isocenter shift of 8 cm. Patients with a PTV extension of more than 36 cm are currently not suited for treatment on a Halcyon unit (Version 2.0).

## Conclusions

We implemented a treatment technique with a tangential beam arrangement for whole breast irradiation. Plan quality was equally good on Halcyon and Synergy Agility machines. Monitor units increased on average by 50% for Halcyon plans due to the sliding window technique. A significantly lower dose to the contralateral breast was measured for the Halcyon plans due to less interleaf transmission of Halcyon’s dual layer MLC and some scattered dose from the collimator system.

## Data Availability

Research data are stored in an institutional repository and will be shared upon request to the corresponding author.

## Abbreviations

3D CRT: Three-dimensional conformal radiotherapy
BEV: Beams eye view
CBCT: Cone beam computer tomography
CTV: Clinical target volume
DIBH: Deep inspiration breath-hold
DVH: Dose volume histogram
FFF: Flattening filter free
HI: Homogeneity index
IGRT: Image guided radiation therapy
IMRT: Intensity modulated radiation therapy
MLC: Multileaf collimator
MU: Monitor unit
OAR: Organ at risk
PTV: Planning target volume
QA: Quality assurance
SD: Standard deviation
VMAT: Volumetric modulated arc therapy
